# MicroRNA-Mediated Hormonal Control of Fruit Morphology

**DOI:** 10.3390/plants15010167

**Published:** 2026-01-05

**Authors:** Kanghua Du, Da Zhang, Weiwu Lv, Guangping Chen, Lingfeng Bao, Xiaomei Li, Wanfu Mu, Zhong Dan

**Affiliations:** Institute of Tropical Eco-Agriculture Science, Yunnan Academy of Agricultural Sciences, Chuxiong 651300, China

**Keywords:** cucumber, microRNA, fruit development, temperature stress

## Abstract

Fruit morphogenesis represents a complex biological process resulting from the interactions among transcriptional regulation, hormone signaling, and environmental factors. MicroRNA (miRNAs) have been recognized recently as key genetic and epigenetic regulators in various plants, and they play critical roles in the regulation of diverse processes in response to endogenous developmental signals and external environmental cues, respectively. Recently, miRNA-mediated regulation mechanisms have also been extensively in horticulture plants, many novel mechanisms unveiled. Compared with model plants and field crops, miRNAs exhibit greater complexity and unique regulatory characteristics in governing fruit development in horticultural crops. Integrating the latest research, this review explores the roles of conserved miRNAs across multiple horticulture crops and synthesizes their regulatory networks in conjunction with phytohormones and transcription factors in governing fruit development, morphogenesis, and stress responses. It highlights the dual role of plant miRNAs under temperature stress, coordinating temperature adaptation, and fruit developmental plasticity through hormones and transcription factor networks. This review discusses the challenges and future prospects of utilizing this complex but promising epigenetic mechanism for crop improvement to cope with climate change.

## 1. Introduction

As a pivotal trait determining the commercial value of horticultural crops, fruit morphology serves as a critical interface between biological development and agricultural economics, where geometric precision directly translates to commercial competitiveness in the global supply chain [1]. From a consumer perspective, optimal morphological characteristics, including geometric symmetry, vibrant coloration, and surface glossiness, constitute fundamental visual parameters governing market acceptance. Standardization of fruit morphology significantly extends shelf life, and enhances efficiency in mechanical harvesting, packaging and transport logistics, whereas malformed fruits incur supply chain loss rates as high as 22% [2].

Fruit morphology is primarily governed by the coordination control of cell division and expansion that are fundamental cellular processes underlying development in all plant organs [3]. The genetic basis of fruit morphology is complex, reflecting its nature as a quantitative inherited trait governed by multiple genes. As established models for Solanaceae, Cucurbitaceae, and Rosaceae, tomato, cucumber, and peach have been widely studied to dissect fruit morphogenesis, respectively. *FW*, *SUN*, *OVATE*, *IC*/*FAS*, *SF* and *HDC1*, key loci regulating fruit morphogenesis, were identified through quantitative trait loci (*QTLs*) fine-mapping [4,5,6]. Understanding the genetic basis of fruit morphology is crucial for breeding cultivars adapted to diverse ecological niches. However, major obstacle in deciphering the genetic architecture of fruit morphology is the masking effect of major loci, which often conceals the action of minor-effect *QTLs* and impedes a fuller understanding of the regulatory network controlling fruit development [7]. To dissect the complex regulatory network governing fruit morphogenesis, multi-omics approaches, including transcriptomics and small RNA profiling, must be integrated with classical quantitative genetics.

On the other hand, genome-wide and transcriptomic analyses have revealed that over 50% of the genome is transcribed in recent years. However, only 1.5% of these transcripts are translated into proteins, while the majority comprise non-coding RNAs (ncRNAs) that lack protein-coding potential [3]. miRNAs are a type of non-coding small RNA, but much of our knowledge regarding miRNAs comes from model plants like Arabidopsis. Since the Nobel Prize-winning discovery of miRNA’s role in gene regulation, extensive identification of miRNAs across diverse fruit species has been achieved through integrated approaches encompassing bioinformation prediction, high-throughput sequencing and experimental validation [8,9,10,11,12]. Research on the role of miRNAs—which mediate posttranscriptional gene regulation in eukaryotes and modulate critical process including meristem function, organ morphogenesis and stress response—pioneered model plants. This foundation is now guiding a progressive expansion of inquiry non-model systems, including a group of horticultural plants [13,14]. It is precisely because of the function roles of miRNAs in fruits that crops exhibit notable mechanistic complexity and species-specific regulatory networks that differ from model plants and field crops that the role of miRNAs in fruit plants is worthy of our further research and exploration, expanding and deepening people’s understanding and thinking about this specific epigenetic mechanism.

This review examines the role of miRNAs in fruit morphogenesis, emphasizing miRNA-mediated hormone synthesis and signal transduction pathways that govern key agronomic traits such as fruit size, shape and parthenocarpy. This review also explores the roles of gene regulatory network comprising miRNAs, plant hormones, and transcription factors in fruit development under temperature stress. Based on recent research gaps and future directions to guide improved fruit quality and stress-resistant breeding.

## 2. MicroRNA-Mediated Regulation of Hormone Pathways in Fruit Development

### 2.1. Evolutionary Conservation and Functional Divergence of miRNAs in Fruit Development

To date, a large number of miRNAs have been identified and cataloged, highlighting their essential functions in numerous metabolic processes [15]. Many previous studies have shown that miRNAs are evolutionarily conserved across all significant plant lineages. These conserved miRNAs have similar effects on their target genes, and the synthetic pathway and target genes of the same miRNAs family are always the same in various plants [16,17,18]. In fruit plants, evolutionarily conserved miRNAs govern developmental patterning, including size and shape. In contrast, species-specific miRNAs tend to modulate secondary metabolic pathways that are often related to pigmentation, aroma production, sugar accumulation, and resistance [19]. However, current miRNAs research remains largely confined to a few model plants and economically important fruits crops, with limited functional characterization in non-model plants, such as cucumber, watermelon, lychee and longan, thereby constraining a comprehensive understanding of fruit morphogenesis. Therefore, this review aims to synthesize current knowledge on key fruit-related miRNAs and their functions, with a focus on the regulatory networks underlying fruit morphogenesis.

**Figure 1:**
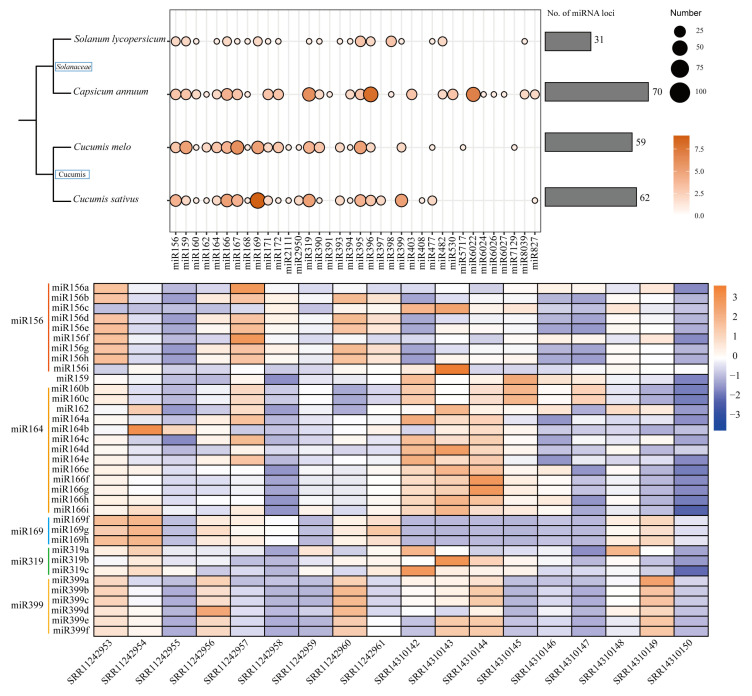
Conservation of miRNAs in fruit. miRNAs among four common fruit and vegetable species, which are derived from NCBI database. The scatter plot on the up shows the number of miRNAs loci in each species. The bar graph on the down shows five miRNAs families which involved in the signaling pathways related to hormone synthesis and metabolic pathways in fruit development. Lowercase letters represent a specific member of the conservation miRNA family.

In this review, we used the ‘One step sRNAminer’ program in the sRNAminer software (v1.1.2) [20] to analyze the sRNA sequencing data of fruit from several fruit and vegetable crops such as tomato, cucumber, melon, and pepper based on NCBI public database. A total of 3950 miRNAs involved in fruit development. The target genes of the screened miRNAs were predicted, and the target genes were analyzed for GO/KEGG, focusing on the signaling pathways related to hormone synthesis and metabolic pathways by the ‘Target prediction’ program in the sRNAmiber software. Comparing the seed sequence of miRNAs, we found conserved miRNAs that belong to 5 miRNAs family, namely *miR156*, *miR164*, *miR169*, *miR319* and *miR399*, which were involved in regulating hormone synthesis and metabolism (Figure 1).

In general, plant miRNAs are commonly classified into three categories: the highly conserved across angiosperms, moderately conserved within certain lineages or families, and those that are species-specific [21]. This review focuses on microRNAs associated with hormone signaling pathways in fruits of diverse horticultural crops. These miRNAs represent conserved regulators of fundamental processes, including plant growth and development, fruit ripening, and quality formation. Despite high sequence conservation, miRNAs families exhibit substantial interspecies divergence in copy number, spatiotemporal expression patterns, and regulatory networks, reflecting their adaptive evolution under natural selection.

The *miR156*, an abundant and highly conserved miRNA family member in plants, functions as a key regulator of fruit development by repressing SPL/SBP-box transcription factors. However, the *miR156-SPL*/*SBP-box* module exhibits substantial functional diversity across crop species. Despite its evolutionary conservation, the *miR156-SPL*/*SBP* module exhibits remarkable functional diversification across crop species. This is well illustrated in tomato, where it induces parthenocarpy through the *GA* pathway. Moreover, the same module regulates later processes, orchestrating fruit color change via ethylene signaling and maintains meristematic identity during early fruit development [22,23,24]. The conserved *miR164* family governs fruit ripening by fine-tuning the expression of *NAC* transcription factors. Base changes in the non-cleavage sites of miR164 have occurred in crops such as wheat (*Triticum aestivum* L.), Gossypium hirsutum and Xanthoceras sorbifolium. Consequently, an expanded repertoire of transcription factors, including *PSK*, *MAPK*, *CRKs*, *NEK*, and *HSF*, became susceptible to *miR164* targeting, among others, facilitating the emergence of novel regulatory architectures and functional diversification [25].

Current research has predominantly focused on linear regulatory relationships between conserved miRNAs and their target genes. In contrast, the crosstalk, feedback loops, and network-level interactions between miRNAs and hormone signaling pathways remain poorly understood.

### 2.2. miRNA–Hormone Interplay During Fruit Development: Crosstalk, Feedback Loops, and Regulatory Networks

Fruit development comprises overlapping phases of cell division, expansion and differentiation, culminating in maturation and ripening. Plant hormones, including auxin, gibberellin (*GA*), cytokinin (*CK*), ethylene, and abscisic acid (*ABA*), orchestrate fruit development through the regulation of cell division and expansion. Recent studies demonstrate that miRNAs fine-tune fruit development by modulating key regulators of hormone synthesis, metabolism, and signaling. For instance, miRNAs directly target hormone signaling components, including auxin response factors (*ARFs*), *DELLA* protein, cytokinin oxidases/dehydroxylases (*CKXs*), and ethylene biosynthetic (e.g., *ACS* and *ACO*), to fine-tune hormonal homeostasis and downstream responses (Table 1). In auxin signaling, *miR160* and *miR167* target *ARF* transcription factors to regulate fruit cell division and shape [26]. Silencing *miR160* via short tandem target mimics (*STTM*) elevates *ARF* expression and promotes elongated pear-shaped fruit development in tomato [27]. Furthermore, *miR393* targets the auxin receptor *TIR1*/*AFB2*, and *miR319* represses *TCP* transcription factor, collectively maintaining auxin homeostasis and regulating cell proliferation and fruit enlargement [28]. *MiR159* regulates fruit morphology by targeting the *GAMYB2* transcription factor, which in turn controls expression of the *GA* biosynthesis gene *GA3ox2*. Overexpression *miR159* impairs *GAMYB* function, reducing fruit size and promoting elongation, whereas its inhibition enhances fruit expansion [29], *miR172* targets *AP2* transcription factor to modulate *GA* biosynthesis and signaling, thereby regulating fruit cell division and expansion. Overexpression of *miR172* promotes siliques enlargement in *Arabidopsis thaliana* but conversely reduces fruit size and weight in tomato and apple [30,31]. Furthermore, it is interesting to note that silencing *miR172* precursors induces the formation of internal secondary fruits in tomato, revealing its key role in early morphological patterning [32]. Similarly, miR166 overexpression induces secondary fruits by suppressing *HD-ZIPIII* transcription factors [33]. These findings suggest that *miR172* and *miR166* coregulate fruit morphology through an interconnected signaling network. In summary, miRNAs form a multifaceted regulatory network that integrates hormonal signals and directs fruit development through targeted transcription factors.

Within regulatory network, miRNAs fine-tune the downstream gene expression by mediating the post-transcription silencing of transcription factors (TFs) through sequence-specific targeting and represents a central mechanism for precise spatiotemporal control of gene expression during plant development and stress responses [7]. Emerging evidence indicates that RNA degradation products can function as regulatory molecules to fine-tune miRNAs levels through multiple mechanisms. These include binding to cis elements within miRNAs precursors or cognate miRNAs genes, as well as modulating miRNAs biogenesis or turnover. Such interactions often give rise to intricate regulatory circuits, including feedback (*FBLs*) and feedforward loops (*FFLs*), which contribute to the dynamic precision of gene regulation [38,39].

These regulatory circuits play essential roles in fruit development by orchestrating key biological processes, including hormone signaling, cell division and expansion, and metabolite accumulation, thereby ensuring precise control over fruit morphogenesis and quality traits. Carvalho A et al. showed that the regulation of *SITCP2*/*LA* by *miR319* is crucial for tomato fruit morphology. The loss of *miR319* led to a premature *SITCP2*/*LA* expression during gynoecium patterning and exhibited elongated ovary and fruit shape [40]. The transcriptional dynamics of *miR319* during fruit development suggesting a possible negative feedback loop between *miR319* and *SlTCP2*/*LA* [41]. Concurrently, a feedback module consisting of *sly-miR167-SlARF8A/B-SlGH3.4* in tomato fruit. *SlARF8A/B* directly suppresses *SlGH3.4* expression to modulate *IAA* amidation, while elevated auxin levels in-duce *miR167* transcription, post-transcriptionally repressing *SlARF8A*/*B* translation and consequently reducing *IAA* accumulation. Ultimately, forming a feedback loop to maintain auxin homeostasis during the development of locular tissues [35]. Beyond their roles in fruit morphogenesis, regulatory circuits governing ripening and quality formation have been characterized in crop species, such as strawberries and apple [42,43]. The functional characterization of miRNA-mediated regulatory circuits in horticultural crops remains limited, primarily due to constraints in genetic transformation. This knowledge gap is particularly evident in networks involving conserved miRNAs, phytohormones, and transcription factors across diverse crop species.

**Figure 2:**
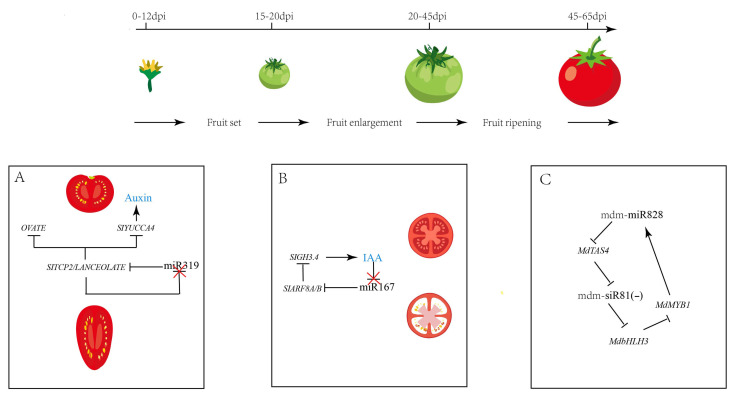
Roles of miRNA–TF loops during fruit developmental. (**A**) A hypothetical model depicting how a regulatory module integrating *miR319*-targeted *SlTCP2*/*LANCEOLATE*, the *OVATE* protein, and auxin signaling coordinates gynoecium patterning to ultimately establish fruit shape. (**B**) A self-correcting feedback module, comprising sly-miR167, *SlARF8A*/*B*, and *SlGH3.4*, maintains auxin homeostasis during tomato locule development through a negative feedback loop to precisely control active auxin levels. (**C**) A model depicting a mdm-*miR828*-*MYB* transcription factor regulatory axis that forms a feedback circuit to maintain anthocyanin homeostasis and prevent over-accumulation in the apple fruit peel.

These regulatory circuits ensure robust execution of the developmental program during fruit ontogeny (Figure 2), while simultaneously integrating internal and external cues, such as hormonal signals, light, and temperature, to facilitate faithful fruit development and ripening under fluctuating environmental conditions. So, elucidating these miRNA-mediated feedback (*FBL*) and feedforward (*FFL*) loops will provide crucial molecular insights and inform novel biotechnological strategies for precision breeding and fruit quality enhancement in crops.

## 3. MicroRNA-Mediated Regulation of Hormone Pathways in Fruit Development Under Temperature Stress

### 3.1. The Effects of Temperature Stress on Fruit

Temperature stress has become a common abiotic stress that has a negative impact on the growth, development and reproduction of plants and on crop production around the world. According to the *IPPC*, the global average annual temperature will increase at a rate of 1.5 to 5.8 °C, and on average, global yields will fall by 2.5~16% each degree-Celsius increase [44]. Reproductive organs are highly sensitive to environmental factors. In particular, temperature stress affects the biological process of another development, style elongation, pollen release, pollen germination, pollen tube growth, pollen-pistil interaction, fertilization and embryo development, with consequences on fruit set and development [45,46,47,48,49]. During reproductive development, fruit organs display greater susceptibility to temperature stress compared to vegetative tissues. Prolonged temperature stress impairs essential physiological functions, such as transpiration, respiration, and photo-assimilate allocation, thereby restricting both mitotic activity and cellular expansion during the cell expansion phase. These physiological disturbances manifest morphologically as reduced fruit elongation and increased incidence of malformed fruits [50,51]. Furthermore, prolonged heat stress detrimentally impacts fruit nutritional quality, manifesting as marked reductions in essential nutritional components including organic acids, vitamin C, soluble proteins, and soluble sugars [52]. Meanwhile, successive high-temperature stresses on young flower buds often cause multilocular fruits, secondary fruit at blossom end in tomato. In addition, the problem of poor pollination, inhibiting fruit development and significantly increasing the frozen fruit rate will also appear at cold stress [53].

The morphological imprint of temperature stress on fruit development is ultimately a consequence of its profound impact on cellular processes, namely the frequency and orientation of cell divisions. Acting as master signaling hubs, phytohormones not only regulate these cellular events but also mediate the plant’s response to thermal stress [54,55]. The integration of these signals is achieved through a complex regulatory symphony involving transcription factors and miRNAs. These players engage in extensive crosstalk, forming a robust network that is dynamically reprogrammed by temperature fluctuations [56]. Elucidating how this integrated network processes environmental information to guide developmental outcomes remains a central question in understanding fruit phenotypic plasticity.

### 3.2. Regulation of miRNAs During Fruit Development by Temperature Stress

**Figure 3:**
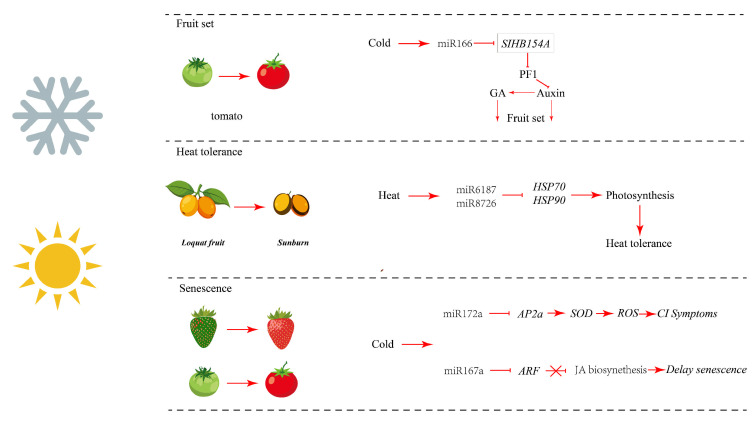
Schematic representation of the regulatory network integrating oxidative signaling, protein homeostasis, and hormone pathways by miRNAs in fruit development underlying temperature stress.

Far from being passive victims of temperature stress, plant fruits actively orchestrate their acclimation through multi-layered miRNAs networks that govern precise transcriptional and post-transcriptional reprogramming. Emerging research indicates that miRNAs function as a key post-transcriptional regulator in coordinating fruit response to temperature stress. By integrating oxidative signaling, protein homeostasis, and hormone pathways, they establish a sophisticated regulatory network that ensures normal fruit development under temperature stress (Figure 3). In response to temperature stress, plant cells initiate cell-autonomous protective mechanisms. In response to heat stress, the specific suppression of *miR6187*/*8726* in loquat fruit enables an enhanced translation of heat shock protein genes. This drives robust HSPs accumulation, offering direct protection to essential organelles including Photosystem II [57]. Complementarily, the activated *miR172a-SIAP2* module in tomato fruit orchestrates a dual-pronged strategy to maintain hardness under cold stress. It concurrently curbs reactive oxygen species (*ROS*) accumulation to minimize oxidative damage and reprograms cell wall metabolism to enhance mechanical strength, thereby collectively preserving the integrity of cellular components [58]. Beyond maintaining cellular homeostasis, developing fruit employs more sophisticated adaptation mechanisms, such as the miRNA-mediated remodeling of fruit morphology via hormone signaling pathways in response to environmental stress. Low temperature-induced *miR166* overexpression in tomato downregulates *SIHB15A*, perturbing the auxin-ethylene crosstalk. This hormonal imbalance reprograms fruit cell proliferation and differentiation, providing a mechanistic basis for the resulting morphological defects [59,60]. In cucumber fruit, miRNAs also demonstrate how temperature signals are transformed into specific morphological changes. The *miR156*/*157* module is used as an environmentally sensitive development regulatory factor. The changes in temperature will drive *miR156*/*157* overexpression, which inversely regulates the expression of levels of the core development regulatory family, such as *SBP-box* transcription factors, which ultimately reprograms the development trajectory of cucumber fruit and influences fruit morphological formation under thermal variation [61,62].

However, it is well established that the integration of environmental stimuli and endogenous signals the orchestration of a broad spectrum of developmental processes, from embryogenesis and fruit set to morphogenesis and stress adaptation [63,64]. Meng et al. identified an auxin-ethylene antagonism governing cucumber parthenocarpy under low temperature, defined by the promotive role of auxin and the inhibitory function of ethylene in fruit development [55]. In tomato plants, prolonged high-temperature stress leads to the formation of malformed fruits by suppressing brassinosteroid biosynthesis, a process mediated by the overexpression of the *SlELF3* gene [65]. These results suggest that plant responses to high temperatures are often tissue and organ specific and are regulated by multiple hormones, regulatory insight into their functions during early morphological development lags considerably behind. Collectively, these findings delineate a hierarchical, miRNA-mediated regulatory network that orchestrates the thermos-responsive development of fruits, spanning from cell-autonomous defenses to systemic hormone-driven remodeling. By precisely influencing the synthesis and signal transduction of hormones, it ultimately regulates fruit morphogenesis. This crucial regulatory chain has not yet been systematically analyzed. The core issue hinges on the multifaceted roles of hormone biosynthesis and signaling genes in linking temperature perception to specific morphological outcomes in developing fruits. The central mechanistic question is whether hormonal dysregulation under temperature stress serves as an upstream signal that triggers downstream miRNAs module dysfunction, leading to aberrant fruit morphogenesis. All these problems need further study.

### 3.3. Regulatory Roles of MicroRNAs in Grafting Systems Underlying Temperature Stress Responses

Grafting technology is one of the most economical and effective means by which to increase yield, improve quality, and enhance the stress resistance/tolerance of horticultural crop plants. It is commonly believed that granted rootstocks and scions maintain their genetic identity, but transcription factors, small interfering RNA (SiRNAs), miRNAs, mRNAs, peptides, and proteins are mobile in the plant vascular system, thereby moving between graft and scions [66,67,68,69]. In graft systems, these mobiles signaling molecules, notable miRNAs, function as long-distance epigenetic modifiers. By trafficking between scion and rootstock, they coordinate transmissible RNA silencing and antagonize DNA methylation, collectively regulating heritable phenotypic variation [70,71,72]. The epigenetic alterations were predominantly enriched in biological processes critical for fruit development, including photosynthesis, oxidative phosphorylation, photosynthetic proteins, and ubiquitin-mediated proteolysis, highlighting their central role in regulating fruit ripening and maturation [72,73].

Recent studies have shown that miRNAs can be mobile, acting as long-distance signaling in graft systems, whereby their translocation from rootstock to scion directly regulates the expression of genes governing critical agronomic traits, such as flowering and fruit morphology. In the context of developmental regulation, the conserved miRNA families *miR166* and *miR396*, identified by their highly specific expression in apricot-peach graft chimeras, have been established as key mobile regulators that govern flowering. The functional analysis indicates that the complex regulatory network formed by the module of *miR396*-*SFH12* and *miR166*-*Bhlh74*, jointly activates the expression of the *FLOWERLOCUST* (FT) gene, thereby synergistically promoting flowering. The results indicate that mobile miRNAs from the rootstock mediate the formation of a multi-layered regulatory network in the scion, enabling the coordinated reprogramming of its core developmental pathways [74]. In regulating fruit size, miRNAs exert more direct control. In the walnut grafting systems, the downregulation of *miR396a* derepresses its target, the *JrGRE4b-JrGIF1a* complex, thereby activating the downstream growth-promoting gene *JrGATA4*, driving scion cell proliferation and ultimately contribute to higher yield potential [75]. Correspondingly, significant alterations in fruit size within pumpkin-watermelon grafts are associated with the reduced expression of *miR159*, *miR164*, and *miR171*, suggesting that a network of interacting miRNAs coordinately regulate this key developmental trait [76]. Clear evidence indicates that graft systems improve scion performance through multiple mechanisms, including enhanced nutrient acquisition, a shifted balance from vegetative to reproductive growth, and the regulation of endogenous hormone levels, which collectively drive fruit enlargement and yield increase [77,78,79]. Unfortunately, the field remains largely descriptive. Most studies are confined to identifying differentially expressed miRNAs and making bioinformation predictions, lacking direct functional validation in specific grafting systems. Furthermore, it is unclear whether rootstock-derived miRNA signals exert stage-specific regulatory functions during fruit development. Consequently, without this spatiotemporal resolution, we cannot construct an accurate map of miRNAs movement and its regulatory logic.

## 4. Conclusions and Future Perspectives

Accumulating evidence has now firmly established that miRNAs play a central regulatory role in determining fruit morphology. Functioning as key post-transcriptional and epigenetic regulators, miRNAs act as a key that integrates hormonal signals and modulates the expression of hormone-related genes. Through this role, they orchestrate complex networks essential for fruit morphogenesis [80]. However, significant knowledge gaps remain. Unlike in module plant and major food crops, the role of miRNAs within the precise regulatory network governing fruit development, particularly their interplay with environmental stress signals, is still largely unexplored. This review synthesizes the morphological and molecular basis of fruit development, with a focus on the complex regulatory networks involving miRNAs, transcription factors, and hormone signals under temperature stress. We propose that hormone signaling acts as a central hub, integrating miRNA-mediated stress responses into developmental outcomes. This is exemplified by the *miR166*-*SIH15A* module, which disrupts auxin-ethylene homeostasis to induce fruit malformation under cold stress. A central question is how hormone signals molecularly integrate miRNA inputs with target gene expression to precisely control processes like fruit cell division, enlargement, and quality formation. Currently, there is a paucity of systematic functional studies to provide a definitive mapping of this regulatory circuitry. Therefore, a prioritized focus on critical phases like flowering and early fruit development is essential for future mechanistic research. Leveraging cutting-edge spatial omics and single-cell transcriptomics will allow for the concurrent capture of miRNAs and mRNA profiles. Generating such dynamic expression maps across tissues and stages is a prerequisite for systematically analyzing how miRNAs serve as core signaling hubs that integrate developmental, hormonal, and environmental cues [81,82].

As discussed in the preceding sections miRNAs have multiple characteristics that give them the potential to play prominent roles during plant growth, development and stress resistance. In this context, the royal road to decipher their roles is a functional approach analyzing the molecular mechanism of abnormal miRNAs function through genetics. Precise functional analysis of individual miRNAs in specific tissues remains challenging. Although CRISPR/Cas9 and RNAi are powerful, their utility in non-model vegetable crops is limited by inefficient transformation. Alternative approaches like *STTM* and *VIGS* often lack robustness and exhibit variable silencing efficiency across tissues, complicating phenotypic interpretation. It is equally important to acknowledge that, even with advances in mechanism understanding, pivotal bottlenecks, such as delivery efficiency, target specificity, and environmental stability, remain to be addressed. Consequently, advancing plant-adapted, spatiotemporally controllable miRNAs technologies, by leveraging intelligent delivery platforms such as nanocarriers, represents a critical frontier for realizing their full application potential.

Furthermore, grafting systems can serve as a powerful platform to overcome the bottleneck of recalcitrant transformation in vegetable crops. A practical strategy involves using easily transformable model plants (e.g., *Nicotiana benthamiana*) or amenable crops within the same family as rootstocks, grafted with the target vegetable scion. Integrating this classical approach with multi-omics analyses can clarify how rootstock-derived mobile miRNAs regulate fruit development under environmental stress. In summary, grafting systems constitute a powerful model system for dissecting the fundamental question of how mobile miRNAs systemically regulate fruit development under environmental stress.

## Figures and Tables

**Table 1:** Mechanisms of miRNA-target modules in regulating fruit development.

miRNA	Direct Target Genes	Evidence Type	Downstream Target Genes	Hormone Crosstalk	Phenotypic Effects	Species	Reference
*miR156*	*SPL 13B*	Predicted	GA biosynthesis genes, *MdKO*, *MdKAO2* and *MdGA20ox*	Regulate *GA* accumulation	Induced parthenocarpy	Tomato	[24]
		Predicted	Histone modification gene, *MdMSI*	Promote *GA* deactivation			
*miR393*	*TIR1*/*AFB2*	Predicted	Undetermined	Maintain the dynamic balance of auxin	Regulate cell proliferation and fruit enlargement	Peach	[28]
		Predicted		Induced ethylene biosynthesis through auxin signaling pathway	Induced fruit ripening	Banana	[34]
*miR160*	*ARF10B*	Validated	Undetermined	Inhibit auxin biosynthesis	Regulate cell division and preserve the fruit morphology	Tomato	[27]
	*ARF8*, *ARF10*, *ARF16*	Validated	Undetermined	Auxin signaling pathway	Regulate the formation of pericarp cell layers	Tomato	[26]
*miR167*	*ARF8B*	Validated	Acyl acid amino synthetase gene, *SlGH3.4*	Disruption of auxin balance	Defective locular and placental tissues in tomato fruit	Tomato	[35]
*miR172*	*AP2*	Validated	Ethylene synthesis gene, *ACS2*, *ACS4*, *ACO1*	Promote ethylene synthesis	Regulates fruit ripening	Tomato	[32]
*miR159*	*GAMYB*	Validated	*GA* biosynthesis gene *GA3ox2*	Regulate *GA* accumulation	Influencing fruit morphology	Tomato	[29]
		Predicted	Undetermined	*GA* signaling pathway	Induced parthenocarpy	Grape	[36]
*miR396*	*GRFs*	Validated	Undetermined	Regulate auxin biosynthesis	Inhibited fruit enlargement	Tomato	[37]

## Data Availability

The RNA-seq datasets used in this study were retrieved from the NCBI public database (SRA accession: PRJNA624184, PRJNA438371, PRJNA718887 and PRJNA624184).
